# Physiological and Transcriptomic Analyses Reveal Divergent Responses of Vegetative Growth and Inflorescence Development to Temperature in *Chrysanthemum* × *morifolium*

**DOI:** 10.3390/life16101600

**Published:** 2026-09-24

**Authors:** Qian Zhang, Si-Yu Zhao, Hong Jiang, Mei Nie, Zhong-Yi Yang, Jin-Hui Lv

**Affiliations:** 1 College of Forestry, Shanxi Agricultural University, Jinzhong 030801, China; zqianabc@sxau.edu.cn (Q.Z.); 13231994358@139.com (S.-Y.Z.); m17382981335@163.com (H.J.); nm550847685@126.com (M.N.); msq5936@163.com (Z.-Y.Y.); 2 Shanxi Key Lab. for Modernization of TCVM, College of Forestry, Shanxi Agricultural University, Jinzhong 030801, China

**Keywords:** *Chrysanthemum* × *morifolium*, ambient temperature, floral bud differentiation, physiological response, transcription factors

## Abstract

Temperature strongly affects chrysanthemum flowering, but its stage-specific effects on vegetative and reproductive development remain unclear. Here, the obligate short-day chrysanthemum cultivar *Chrysanthemum* × *morifolium* ‘Zijiao’ was grown under three different day/night temperature regimes. We anatomically identified eight stages of floral induction and differentiation by observing the shoot apical meristem. The 25/16 °C treatment promoted coordinated inflorescence development, with floral bud differentiation completed within 42 days, the greatest number of florets, and the largest floral buds among the treatments. In contrast, the 30/18 °C regime enhanced plant height and internode elongation but delayed late floral bud differentiation. Photosynthetic pigment levels were generally maintained under the two warmer treatments relative to 20/13 °C, whereas carbohydrates, soluble proteins, superoxide dismutase, and peroxidase showed temperature- and stage-dependent changes. Leaf transcriptome profiling after 10 days revealed the differential regulation of hormone signaling, primary metabolism, antioxidant responses, and peroxisome-related pathways. The developmental expression of *heat shock transcription factors A* (*CmHSFA4*), *CmWRKY40*, *CmMYB*, and *CmGATA8* further indicated stage-specific transcriptional responses. T2 and T3 showed significant positive relationships with *CmMYB* and *CmWRKY40* expression, respectively. Thus, under the conditions tested, intermediate temperature favored reproductive development, whereas warmer conditions sustained vegetative growth and acclimation but compromised late floral organogenesis. These findings provide a physiological and transcriptional basis for cultivar-specific temperature management in chrysanthemum production.

## 1. Introduction

Cultivated chrysanthemum (*Chrysanthemum* × *morifolium*) is an economically important horticultural crop widely cultivated for its ornamental, edible, and medicinal value. Floral transition and subsequent capitulum development are major determinants of flowering time, inflorescence architecture, and marketable flower quality. Many commercial chrysanthemum cultivars exhibit an obligate or strong short-day flowering response; photoperiod manipulation is, therefore, widely used to regulate flowering for year-round production. Nevertheless, flowering time and flower quality remain highly sensitive to growth temperature even under inductive short-day conditions. Temperature affects not only the transition from vegetative to reproductive growth but also the subsequent differentiation and expansion of the capitulum, and these responses vary among cultivars and developmental stages [1,2]. In chrysanthemum, high temperatures can repress the expression of the short-day florigen gene in leaves and delay the activation of floral meristem- and floral organ-identity genes at the shoot apex. Consequently, capitulum and floret development may be inhibited even after reproductive transition has occurred [3]. However, previous studies have primarily evaluated temperature effects using phenological endpoints, such as time to flowering and visible-bud or capitulum development [4,5], whereas other studies have examined physiological and molecular responses to acute or severe heat-stress regimes [6,7]. Therefore, the stage-specific effects of horticulturally relevant temperature regimes on floral induction, floral bud differentiation, and late floral organogenesis remain insufficiently characterized.

Successful inflorescence development requires the coordinated regulation of photosynthetic carbon assimilation, assimilate allocation, osmotic adjustment, and cellular redox homeostasis. Chlorophylls and carotenoids (Cars) support light harvesting and photoprotection, whereas starch remobilization and soluble sugar accumulation provide both metabolic substrates and signaling molecules for developing reproductive sinks [8,9]. Soluble proteins contribute to osmotic regulation, enzyme activity, and cellular protection, while reactive oxygen species (ROS) function as developmental signals at controlled concentrations but cause oxidative damage when excessively accumulated [9,10]. Superoxide dismutase (SOD) and peroxidase (POD) are, therefore, pivotal components of the antioxidant system that maintains redox balance during environmental acclimation and reproductive development [11,12]. These physiological traits are strongly influenced by temperature, but their responses may also change as floral bud differentiation proceeds. This distinction is particularly important because vegetative growth, pigment maintenance, and stress acclimation can continue under a suboptimal reproductive temperature even when floral organ formation is impaired. High temperatures have been shown to inhibit photosynthesis in chrysanthemum seedlings [6], but comparatively little is known about how carbon metabolism and antioxidant activity change across anatomically defined stages of chrysanthemum inflorescence development under moderate temperature differences.

At the molecular level, temperature responses are mediated by extensive interactions among phytohormone signaling, stress-response pathways, primary metabolism, and transcriptional regulation. Auxin (AUX), gibberellin (GA), and brassinosteroid (BR) signaling pathways contribute to cell expansion and temperature-dependent stem elongation through components such as *AUX*/*IAA*, *ARF*, *SAUR*, *GID1*, *DELLA*, *BRI1*, and *TCH4* [13,14,15,16]. In contrast, abscisic acid (ABA), ethylene (ETH), jasmonate (JA), mitogen-activated protein kinase (MAPK) signaling, and peroxisome-associated processes contribute to osmotic adjustment, ROS homeostasis, stress acclimation, and the balance between vegetative and reproductive growth [17,18,19,20]. Temperature-responsive transcription factors (TFs), including members of the *heat shock transcription factor* (*HSF*), *WRKY*, *MYB*, and *GATA* families, further connect environmental signaling with antioxidant defense, carbon and nitrogen metabolism, and flowering regulation [21,22,23,24]. Although individual components of these pathways have been investigated in chrysanthemum or model plants, the relationship between early temperature-induced transcriptional reprogramming and subsequent physiological and developmental responses during chrysanthemum floral bud differentiation remains unclear. In particular, it is not known whether a temperature regime that maintains vegetative growth and activates protective responses necessarily favors the completion of normal floral organogenesis.

Field cultivation observations indicate that growth temperature differentially affects vegetative growth and inflorescence development in *C.* × *morifolium*, with an intermediate temperature favoring coordinated floral bud differentiation, whereas warmer conditions promote stem elongation. To determine whether these field observations could be reproduced under controlled conditions and to investigate their developmental, physiological, and transcriptional basis, the obligate short-day ground-cover cultivar ‘Zijiao’ was grown under three day/night temperature regimes. The principal stages of floral induction and differentiation were first anatomically defined by examining the shoot apical meristem. Developmental duration, plant morphology, photosynthetic pigments, carbohydrates, soluble proteins, and antioxidant enzyme activity were subsequently evaluated throughout floral bud differentiation. Early in this stage, transcriptome profiling combined with the developmental expression analysis of selected temperature-responsive transcription factors was also performed to identify physiological and transcriptional signatures associated with the contrasting effects of temperature on vegetative growth and reproductive development. The results provide a stage-resolved framework for optimizing temperature management in chrysanthemum production and identify candidate regulatory pathways for future functional investigation.

## 2. Materials and Methods

### 2.1. Plant Materials and Temperature Treatments

Ground-cover *C.* × *morifolium* ‘Zijiao’, maintained at Shanxi Agricultural University (Tai Gu district, Jinzhong, China), is an obligate-short-day cultivar with high ornamental value. Healthy and vigorous shoots of ‘Zijiao’ were selected and propagated by using cuttings, which were briefly immersed in a rooting solution and subsequently rooted in loose sandy loam soil. The rooted plants were transplanted into the nursery of Shanxi Agricultural University on May 1 of the same year and grown under open-field conditions. In May of the following year, after natural overwintering, uniform two-year-old plants with similar growth vigor were selected and transplanted individually into plastic pots (20 cm in diameter × 25 cm in height) containing a mixture of commercial nutrient substrate, sand, and loam in a volume ratio of 1:1:1.

Based on previous studies [25] and the preliminary experimental data collected by our research group, ‘Zijiao’ exhibited obvious differential responses to three temperature treatments: Temperature 1 (20/13 °C, T1), Temperature 2 (25/16 °C, T2), and Temperature 3 (30/18 °C, T3). After a 20 d establishment period following potting, the plants were randomly assigned to the three temperature treatments. Each temperature treatment was independently replicated in three growth chambers, resulting in a total of nine growth chambers, with an equal number of plants placed in each chamber. Pots were randomly positioned within each chamber, with a spacing of approximately 15 cm between adjacent plants.

During the temperature treatments, plants were maintained under a 16 h light/8 h dark photoperiod at a relative humidity of approximately 70%. White light was supplied at a photosynthetic photon flux density (PPFD) of approximately 300 μmol photons m^−2^ s^−1^. The actual chamber temperatures were maintained within ±2 °C of the designated temperature setpoints. Plants were irrigated as needed to maintain the substrate water content at approximately 60% and were supplied with a dilute liquid fertilizer every 10–15 d. Plants were maintained under the designated temperature regimes for 40 d and were subsequently returned to field conditions for further observation. Air temperature following plant relocation to the field is provided in Figure S1.

### 2.2. Anatomical Observation of Floral Bud Differentiation

The anatomical structures of the shoot apical meristem (SAM) were investigated to identify the principal stages of floral induction and differentiation under each temperature regime. The stem apices and visible floral buds were collected from 10 randomly selected plants every two days until floral color became visible (Figure 1). Samples were fixed in formalin–acetic acid–alcohol liquid (70% ethyl alcohol–glacial acetic acid–methanol; 18:1:1) for permanent paraffin sectioning. Preparation followed a published procedure with minor adjustments [26]. Sections were stained with hematoxylin, dehydrated with graded ethanol, cleared, and infiltrated with paraffin three times at 55 °C.

### 2.3. Morphological and Developmental Measurements

Plant height and crown width were recorded from the onset of treatment until the completion of floral bud differentiation. For each treatment, 10 individual plants were randomly selected. Internode length was determined from the fourth to the sixth internodes below the shoot apex, and leaf length and width were measured on fully expanded leaves from the middle portion of each plant. Flower diameter and the number of flowers per plant were recorded at full bloom, defined as 50% of flowering.

### 2.4. Physiological and Biochemical Measurements

Fully expanded leaves from the middle portion of each plant were collected between 9:00 and 10:00 every two days. At each sampling time, three independent biological replicates were collected for each treatment. Each biological replicate consisted of 30 fully expanded leaves collected from three randomly selected plants. For pigment analysis, tissues were extracted in 96% (*v*/*v*) ethanol in darkness for approximately 24 h. Absorbance at 470, 649, and 665 nm was measured with a spectrophotometer (Thermo Fisher Scientific, Shanghai, China), and Chl a, Chl b, carotenoid, and total chlorophyll contents were calculated according to Lichtenthaler and Wellbum [27].

The antioxidant activity of the SOD and POD enzymes was measured as previously described [28,29]. Soluble sugars and starch were quantified with the anthrone colorimetric assay [30,31], with absorbance recorded at 630 and 620 nm, respectively. Soluble protein was measured with a minor modification of Marion’s method [32]. Briefly, 0.1 mL of crude extract was combined with 0.9 mL of distilled water and 5 mL of Coomassie Brilliant Blue reagent, and absorbance was read at 595 nm against a reagent blank. Soluble protein content was determined as (mg·g^−1^ FW) = (C × VT)/(W × VS × 10^3^), where C represents the value derived from the standard curve, VT is the total extract volume, W is the fresh sample mass, and VS is the aliquot volume used in the assay.

### 2.5. Transcriptome Sequencing and Bioinformatic Analysis

Fully expanded leaves located around the third-to-fourth internodes were harvested on treatment day 10 (I2 stage) and immediately frozen in liquid nitrogen. Three independent biological replicates were prepared for each temperature. Each biological replicate consisted of 30 fully expanded leaves collected from three randomly selected plants. Total RNA was extracted, and its quality was assessed with a NanoDrop 1000 (Thermo Fisher Scientific, Waltham, MA, USA). Libraries were generated using NEB Next^®^Ultra™ RNA Library Prep Kit for Illumina^®^ (NEB, Ipswich, MA, USA) following the manufacturer’s instructions. Briefly, poly(A)^+^ mRNA was isolated from total RNA and fragmented, followed by reverse transcription and second-strand cDNA synthesis. The resulting cDNA fragments underwent end repair, adenylation, adaptor ligation, and size selection. After enzymatic treatment, the libraries were PCR-amplified and purified. Then, PCR was performed with Phusion High-Fidelity DNA polymerase, Universal PCR primers, and Index (X) Primer. Indexed libraries were clustered using a cBot Cluster Generation System with the TruSeq PE Cluster Kit v3-cBot-HS (Illumina, Inc., San Diego, CA, USA) and were sequenced on an Illumina Hiseq 2000 platform, and paired-end reads were generated. Raw reads were subjected to quality filtering to remove adaptor-containing reads, reads with excessive poly-N sequences, and low-quality reads. According to the previously reported criteria and descriptions for sequencing-data quality control [33], the Phred quality score (Q-score) was employed as an indicator of sequencing read accuracy; it is defined as Q = −10 × log_10_(P), where P denotes the probability of incorrect base assignment. A higher Q-score reflects improved sequencing accuracy, with Q20 and Q30 corresponding to approximately 99% and 99.9% base-calling accuracy, respectively. Q30 and GC content were evaluated for each library, and the corresponding statistics are provided in Table S6. The reads were further de novo assembled using Trinity v2.14.0., with min_kmer_cov being set to 2 and all other parameters being left at default values. Transcript clustering and redundancy reduction were performed using TGICL v2.1 and MMseqs2 v13.45111 to generate a non-redundant unigene set. The sequences were further processed with a bioinformatics pipeline tool, the BMKCloud (www.biocloud.net) online platform. For each biological replicate, clean reads were mapped back to the assembled transcriptome, and transcript abundance was quantified using RSEM v1.2.19 with the parameters -a -m 200. Because a reference-free de novo assembly was used, a reference-genome mapping rate was not applicable. Q20 and read-back mapping-rate statistics were not available from the retained analysis outputs. After de novo assembly and quality control, clean reads were annotated against the NCBI non-redundant protein database (NR), Swiss-Prot, EggNOG, Gene Ontology (GO), and Kyoto (KEGG) databases using DIAMOND v2.0.4 with an E-value cutoff of 1 × 10^−5^. Protein-domain annotation against the Pfam database was performed using HMMER v3.1b2 with an E-value threshold of 1 × 10^−10^. DESeq2 v1.30.1 was used to identify differentially expressed genes (DEGs) at a false discovery rate (FDR) < 0.01 and a fold change (FC) ≥ 2 [34]. A twofold expression difference is expressed as∣log_2_ FC∣ ≥ 1, with log_2_ FC ≥ 1 indicating upregulation and log_2_ FC ≤ −1 indicating downregulation. Temporal expression patterns were analyzed in R v4.1.3 using the Mfuzz package, v2.54.0. Genes with similar expression trajectories were grouped using fuzzy c-means clustering, and the degree of the association of each gene with a given cluster was assessed using its membership coefficient. Functional enrichment analysis was performed using the R package clusterProfiler, v4.4.4. The *p*-values obtained from multiple enrichment tests were corrected for multiple comparisons using the false discovery rate (FDR) procedure. KEGG pathways with an FDR-adjusted *p*-value < 0.10 were considered significantly enriched.

### 2.6. Developmental Expression Analysis of Candidate Transcription Factors

Four candidate temperature-responsive transcription factors were profiled across floral induction and floral bud differentiation during and after the different temperature treatments using quantitative real-time PCR (RT-qPCR). Mature leaves at the 3rd and 4th nodes were collected from plants at the corresponding developmental stages under each temperature treatment. Three independent biological replicates were collected for each treatment and developmental stage, with each biological replicate consisting of leaves collected from one independently grown plant.

Total RNA was extracted from each sample using RNA extraction kits (Aidlab Biotechnologies Co., Ltd., Beijing, China). RNA integrity was assessed with agarose gel electrophoresis, and RNA purity and concentration were determined using a NanoDrop 2000 c spectrophotometer (NanoDrop, Waltham, MA, USA). RNA samples A260/A280 1.8–2.0 were used for reverse transcription. A total of 1 μg of RNA was reverse-transcribed using the PrimeScript RT Reagent Kit (Takara Biotechnology (Dalian) Co., Ltd., Dalian, China) according to the manufacturer’s instructions.

Reactions were performed with SYBR Premix Ex Taq on a Bio-Rad CFX96 Touch system (Bio-Rad Laboratories, Inc., Hercules, CA, USA) following Wang et al.’s procedure [35]. The reaction volume and cycling conditions are provided in Tables S1 and S2, respectively. A melting-curve analysis was subsequently performed from 55 °C to 62 °C to verify amplification specificity. *Actin* was used as the internal reference gene based on its previous application in RT-qPCR analyses of chrysanthemum under temperature treatments and during developmental processes [36,37]. The amplification efficiencies (E) ranged from 92.1% to 115.9%, with coefficients of determination (R^2^) ranging from 0.992 to 0.998 (Table S3). A single melting peak was observed for each primer pair, and agarose gel analysis further confirmed the presence of a single amplification product of the expected size (Figure S2), supporting primer specificity. No second candidate reference gene was experimentally evaluated in the present study. Therefore, relative-expression normalization was based on *Actin* alone. Three technical qPCR replicates were performed for each biological replicate, and the Ct values were averaged before relative transcript abundance was calculated using the 2^−ΔΔCt^ method [38]. Statistical analyses were conducted using the three biological replicates (*n* = 3), rather than individual technical replicates.

### 2.7. The Correlation Relationship Analysis

The correlation relationships of the growth phenotypic characteristics, the physiological and biochemical parameters, and the expression profiles of the candidate temperature-responsive TFs during floral bud differentiation during and after different temperatures were analyzed using IBM SPSS Statistics 20.0. Advanced Cor link and Mantel tests were performed using R v3.6.2 (l).

### 2.8. Data Analysis

Statistical analyses were performed using IBM SPSS Statistics. For variables measured across multiple developmental stages, temperature treatment, developmental stage, and the temperature × developmental-stage interaction were evaluated using a factorial statistical framework. When a significant temperature × developmental-stage interaction was detected, simple-effect analyses were subsequently performed to compare temperature treatments within each developmental stage and developmental stages within each temperature treatment. For variables repeatedly measured on the same individual plants, a repeated-measures ANOVA was used, with developmental stage as the within-subject factor and temperature treatment as the between-subject factor. Post hoc multiple comparisons were performed using Duncan’s multiple range test, with *p* < 0.05 being considered statistically significant. Figures were prepared using GraphPad Prism 9.5 and Adobe Photoshop CS5.

## 3. Results

### 3.1. The Identification of Floral Bud Differentiation Stages in ‘Zijiao’

To determine how temperature affects chrysanthemum flowering, we first anatomically defined the key stages of floral bud differentiation by observing the shoot apical meristem (SAM). In the vegetative juvenile (VJ) stage, the terminal bud continued to grow vegetatively, with the SAM being smaller and slightly protruding. The SAM was wrapped by the differentiated leaf primordia, presenting in different sizes and asymmetry patterns (Figure 2a). Then, the floral development in ‘Zijiao’ was separated into eight stages distributed across inflorescence differentiation (I1–I3) stages (Figure 2b–d) and floret differentiation (F1–F5) stages (Figure 2e–i). Firstly, the vegetative SAM enlarged and acquired a hemispherical shape in the inflorescence primordium initiation (I1) stage (Figure 2b). Subsequently, the transitioning SAM continued to expand and became a round and smooth protrusion. The involucre primordia curving inward differentiated around the inflorescence meristem in the I2 stage (Figure 2c). At the end of the differentiation of the involucre scales (I3 stage), the SAM tended to flatten, and the involucre scales continued to grow, wrapping the SAM (Figure 2d). In the initial floret primordium differentiation (F1) stage, only the lower part differentiated into the floret primordia, appearing as two–three layers of round granular protrusions (Figure 2e), while the middle and upper parts differentiated at the end of the floret primordium differentiation (F2) stage (Figure 2f). Similarly, in the corolla formation stage (F3), the floret developed initially in the lower part (initial stage; Figure 2g) and then in the middle and upper parts (middle and final stages; F4, F5) (Figure 2h,i) of the inflorescence meristem, i.e., from the base upward.

### 3.2. The Effects of Temperature on ‘Zijiao’ Floral Bud Differentiation and Plant Morphology

Compared with T1, treatments T2 and T3 shortened the overall duration of floral bud differentiation duration in ‘Zijiao’ to different extents (Figure 2j, Table S4). T2 had the greatest promotive effect, with floral bud differentiation completed within 42 d, which was 27 and 30 d earlier compared with plants treated with T1 (72 d) and T3 (69 d), respectively. This effect of treatment T2 was primarily attributed to rapid differentiation in the F1 and F4 stages. Although T3 modestly reduced the total duration relative to T1, it substantially extended the I1, I3 and F4 stages. Floral bud differentiation proceeded most rapidly during the early stage of floral bud development under treatment T1, with visible floral buds being first detected at the end of I3; in contrast, the first buds under T2 and T3 were observed at the beginning of F1 (Figure 3). Repeated-measures ANOVA showed significant effects of developmental stage and temperature treatment, with a significant temperature × developmental-stage interaction (Table S5). The average floral bud diameter was generally the greatest both during and after treatment T2 during late differentiation.

Based on the fitted growth curves, plant height increased rapidly from the VJ stage to 10d after treatment (DAT; I2 stage), and T3-treated plants were taller than those treated with T1 and T2 at corresponding time points both during and after treatment (Figure 4a,b). Crown width increased the most rapidly within 5 DAT. T1 plants generally showed larger crown width, whereas T2 and T3 plants had similar crown width upon floral bud differentiation completion. Among the three treatments, T2-treated ‘Zijiao’ plants produced the greatest number of flowers, while T3 produced the longest internodes. Leaf length and width did not differ significantly among temperature treatments.

### 3.3. The Effects of Temperature on Chlorophyll Pigments During Flowering

Factorial ANOVA showed significant effects of temperature treatment and developmental stage on Chl a, Chl b, carotenoid, and total chlorophyll contents. Significant temperature × developmental-stage interactions were also detected for all four pigment traits (Table S5), indicating that the magnitude of the temperature response varied across floral bud developmental stages. The contents of chlorophyll a (Chl a), chlorophyll b (Chl b), carotenoids (Car), and total chlorophyll showed broadly similar change patterns during and after T2 and T3, increasing during inflorescence differentiation and peaking in F1 (Figure 5). During and after T1, pigment contents showed relatively moderate fluctuations but also reached their highest levels in the F1 stage and the lowest values in the I1 stage. At comparable stages, T1 generally showed lower pigment contents than the warmer treatments, except for the VJ stage. Chl a and total chlorophyll were the highest under T2 from the I2 to F4 stages. However, among the three treatments, Car content during and after T3 was the highest from I1 to F5 stage during floral bud differentiation, while Chl b contents were was the highest after the F1 stage.

### 3.4. The Effects of Temperature on Osmotic Adjustment and Antioxidant Defense

Temperature treatment and developmental stage significantly affected starch, soluble sugar, soluble protein, SOD activity, and POD activity (Table S5). Significant temperature × developmental-stage interactions were detected for soluble sugar, soluble protein, and POD activity, whereas the interactions for starch content and SOD activity were not significant. Starch content declined continuously in all treatments until the F1 stage (Figure 6), with T1 retaining higher starch content than T2 and T3 at corresponding stages. Soluble sugar and soluble protein contents showed fluctuating but broadly similar change patterns during and after distinct temperature treatments, and both peaked in the I1 stage. After the I2 stage, soluble sugar content was consistently the highest during and after T2 among the three treatments. Soluble protein content decreased with the increase in temperature before the I1 stage, whilst from the I3 to F4 stages, it was generally lower under T2 than during and after T1 and T3.

SOD activity increased from VJ to F1 and subsequently declined (Figure 6). T3 consistently showed the highest activity at matched developmental stages. POD activity displayed a more variable pattern, initially decreasing and then increasing to a peak around the I2 stage before declining at later stages during and after T1 and T3.

### 3.5. Transcriptomic Responses to Temperature During Floral Bud Differentiation

Leaf samples were subjected to RNA sequencing to characterize the early molecular response underlying temperature-dependent floral development. Following sequencing quality control, 72.33 Gb of clean data was generated. De novo assembly generated 199,184 transcripts and 75,808 unigenes, with N50 lengths of 1302 and 1255 bp, respectively (Table S7). The T2 exhibited relatively good reproducibility, while some intra-group variation was observed in the T1 and T3 (Figure S3). A total of 497, 1855, and 355 DEGs were identified in the T1 vs. T2, T1 vs. T3, and T2 vs. T3 comparisons, respectively (Figure 7a). Across the pairwise comparisons, 606 DEGs were shared between at least two, and 20 were common to all three. The number of DEGs increased with the temperature difference between treatments. In the T1 vs. T3 comparison, 1426 DEGs were upregulated, and 429 were downregulated (Figure 7a,b). With Mfuzz clustering, we grouped the DEGs into six expression profiles across the temperature gradient, with clusters 1 and 6 containing the largest numbers of genes (Figure 7c).

KEGG enrichment analysis showed that the DEGs were mainly associated with genetic information processing, environmental information processing, and metabolism (Figure 7d). Within the latter two categories, plant hormone signal transduction, MAPK signaling, and starch and sucrose metabolism contained the largest numbers of annotated DEGs. Carotenoid biosynthesis was also enriched in all three pairwise comparisons.

### 3.6. Temperature-Responsive Genes Involved in Phytohormone Signaling

Numerous DEGs involved in temperature-dependent flowering were identified in phytohormone signaling pathways (Figure 8). In the auxin pathway, *AUX1*, *AUX*/*IAA*, and *auxin response factor* (*ARF*) homologs were downregulated with the increase in temperature, whereas two SAUR genes were upregulated. Six cytokinin-related DEGs were identified; only one type-B *ARR* (TRYNITI_DN25554_c0_g1) and one type-A *ARR* (TRYNITI_DN19852_c0_g1) showed progressive induction with temperature. All detected GA-signaling DEGs, including one *Gibberellin-Insensitive Dwarf 1* (*GID1*) and five *DELLA* homologs, were the most highly expressed under T3. Similarly, *brassinosteroid insensitive 1* (*BRI1*), *BAK1- associated receptor kinase 1* (*BAK1*), and *TCH4* of the BR signaling pathway showed increased expression, but only one *BRI1* (TRYNITI_DN9117_c0_g1) was downregulated. In the ethylene pathway, *constitutive triple response 1* (*CTR1*) expression peaked under T2, whereas the expression of *ethylene response (ETR*) and two *ethylene-responsive transcription factor1* (*ERF1*) homologs increased with temperature. Three *JAZ* genes in the JA pathway were also induced by the increase in temperature.

### 3.7. Temperature-Responsive Genes Associated with Physiological Regulation

Six DEG families were associated with carotenoid biosynthesis, and most showed their highest expression under T2 (Figure 9). The MAPK pathway contained upregulated components related to ROS and stress signaling, including *mitogen-activated protein kinase kinase 4/5* (*MKK4*/*5*), *MPK3*/*6*, and *mitogen-activated protein kinase kinase kinase* (*MEKK1*), as well as six *WRKY* genes. ABA-related *PYR*/*PYL* and *MAPK* transcripts also increased with temperature (Figure 8e and Figure 9). In addition, all three DEGs assigned to the peroxisome pathway were upregulated under warmer conditions.

### 3.8. Identification of Differentially Expressed Transcription Factors

In total, 83 DEGs belonging to 38 transcription-factor families were identified across the three comparisons. The most numerically significant genes were 10 *HSF*, 9 *WRKY*, 8 *homeodomain-like protein* (*HD*), 7 *MYB*, 6 *ERF*, 4 *GATA*, 4 *NIN-like protein* (*NLP*), and 3 *APETALA2* (*AP2*) families (Figure 10a). These transcription factors exhibited three major expression patterns and were either positively or negatively associated with the increase in temperature.

Shared *HSF* genes were found in both T1 vs. T2 and T1 vs. T3, with two increasing (TRYNITI_DN4121_c0_g1, TRYNITI_DN8633_c0_g1) and two decreasing (TRYNITI_DN8905_c0_g1, TRYNITI_DN21062_c0_g1) as temperature rose (Figure 10b). The *WRKY* and *ERF* families also contained common DEGs across these comparisons, and most, including TRINITY_DN1084_c0_g1, TRINITY_DN3963_c0_g1, and TRINITY_DN17604_c0_g1, were induced. Overlapping *MYB* (TRINITY_DN6648_c0_g1), *ERF*(TRINITY_DN9755_c0_g1), and *GATA* genes (TRINITY_DN7152_c0_g1 and TRINITY_DN21717_c0_g1) were additionally observed in T1 vs. T2 and T2 vs. T3, and most showed higher expression at higher temperatures.

### 3.9. Developmental Expression Patterns of Candidate Temperature-Responsive Transcription Factors

Furthermore, TFs that exhibited differential expression among comparison groups and maintained relatively high expression abundance across all temperature treatments were selected for subsequent expression profiling analyses, including TRIYNITY_DN8633_c0_g1 (*CmHSFA4*), TRINITY_DN3963_c0_g1 (*CmWRKY40*), TRINITY_DN6648_c0_g1 (*CmMYB*), and TRINITY_DN21717_c0_g1 (*CmGATA8*), which were selected for subsequent developmental expression profiling (Figure 11a). RT-qPCR validation of these candidate temperature-responsive TFs identified by RNA-seq on day 10 showed complete concordance in the direction of expression change across all comparisons Figure S4. Factorial analysis revealed significant main effects of temperature treatment and developmental stage on the expression of *CmHSFA4*, *CmWRKY40*, *CmMYB*, and *CmGATA8*. Significant temperature × developmental-stage interactions were also detected for all four candidate TFs (Table S8). *CmHSFA4* showed broadly similar developmental patterns among treatments, but its expression increased earlier under T3, peaking in the I2 stage, whereas peaks under T1 and T2 occurred in the I3 stage. *CmWRKY40* was also induced earlier by T3, with pronounced expression in the I2 and F1 stages. Under T2, *CmMYB* was strongly upregulated in I1, and in F3, it exceeded the levels observed under T1 and T3 in the corresponding stages. Under T3, *CmMYB* increased in the I1 and F1 stages. *CmGATA8* transcript abundance was enhanced by higher temperatures and peaked in I1 and F4 under T2 and T3, with the highest expression being detected under T3. Collectively, the significant interaction effects were consistent with the observed shifts in the timing and magnitude of transcriptional peaks among temperature treatments.

The results of the correlational analyses of the morphological, physiological, and biochemical indices, as well as flower characteristics and candidate temperature-responsive TFs, showed that there were significant positive correlations among Chla content, total chloroplast content, SOD and POD activity, and starch content (Figure 11b). T2 and T3 were significantly positively correlated with *CmMYB* and *CmWRKY40* expression, respectively.

## 4. Discussion

The present study demonstrates that the temperature response of *C.* × *morifolium* ‘Zijiao’ was nonlinear, developmentally dependent, and markedly different between vegetative growth and reproductive development stages. Previous studies have shown that the rate of chrysanthemum flowering is optimized within a cultivar-dependent temperature range, whereas temperatures below or above this range can delay flowering, particularly by restricting capitulum development after the formation of the inflorescence meristem [39,40]. Consistent with this concept, among the three temperature treatments, the intermediate regime of 25/16 °C produced the most favorable reproductive phenotype in ‘Zijiao’, with complete floral bud differentiation within 42 d, the greatest number of flowers, and the largest floral buds. However, despite the largest temperature difference between treatments T1 and T3, the total duration of floral bud differentiation tended to be similar. In *Antirrhinum majus*, flowers exposed to low and high temperatures showed comparable quantitative emissions of volatile compounds despite showing distinct transcriptomic profiles [41]. Consistent with the literature results, the physiological and biochemical parameters measured in this study also differed among temperature treatments. Moreover, the transcriptomic analysis of the early temperature response identified the largest numbers of differentially expressed genes in the two pairwise comparisons, further indicating that plants activate substantially different molecular responses under distinct temperature regimes.

Plants grown at 30/18 °C exhibited greater plant height and internode elongation, while floret differentiation was markedly delayed but not prevented. This contrasting response indicates the partial uncoupling of vegetative growth from reproductive development under warmer conditions and suggests that late floral organogenesis is more thermally sensitive than stem elongation or general biomass accumulation. Additionally, in line with previous research in chrysanthemum, elevated temperature restricted capitulum and floret differentiation by delaying the activation of floral organ-identity genes at the shoot apex [3]. The 30/18 °C regime represents a suboptimal reproductive temperature range rather than a lethal or generally growth-inhibiting condition for ‘Zijiao’. Thermal sensitivity and heat-induced flowering delay vary substantially among chrysanthemum genotypes [40].

High temperature can alter chlorophyll and carotenoid accumulation in chrysanthemum and other horticultural crops [42]. In the present study, however, T2 and T3 retained higher Chl a, Chl b, carotenoid, and total chlorophyll contents in the F1 stage, consistent with the response reported by Viljevac Vuletić et al. [43]. Transcriptomic analysis on treatment day 10 identified differential expression of several genes associated with carotenoid biosynthesis, including *PSY*, carotenoid cleavage-related genes, and *NCED*. These transcriptional differences coincided with the variation in pigment contents, although their causal relationship with pigment accumulation remains to be verified. *CYP707A* and several *PYR*/*PYL* receptor homologs were also differentially expressed among temperature treatments, indicating temperature-responsive changes in ABA-associated metabolism and signaling.

Temperature-dependent carbohydrate dynamics help explain the differences in floral bud growth. Starch content gradually decreased from vegetative growth to the F1 stage under all treatments, indicating that stored carbon was mobilized during floral primordium formation and bud enlargement. Starch subsequently increased and was the highest in the F3 stage. Nevertheless, T1 buds enlarged and developed more slowly than T2 buds after the F3 stage, indicating that low temperature reduced sink activity and redirected excess carbon toward storage. T1 and T3 resulted in higher soluble sugar levels in the I1 stage, whereas during the subsequent developmental stage, soluble sugar content was the highest under T2 among the three treatments. Soluble content reached a maximum in T2 in the F3 stage. This pattern aligns with the vigorous enlargement of T2 flower buds in the F3 and F4 stages. Starch is converted into soluble sugars to raise osmotic potential as a response to temperature change [6]. Soluble carbohydrates also act as nutritional substrates and endogenous signals during floral bud differentiation and do not necessarily overaccumulate at optimal temperature [44]. Therefore, the superior floral performance during and after T2 compared with the other two treatments appears to be due to sustained carbon allocation and stronger sink activity during late inflorescence development rather than early sugar accumulation. In contrast, the soluble carbohydrate pool retained in T3 plants might be preferentially diverted to osmotic protection and stress defensive metabolism, instead of being fully channeled into the morphogenesis of intact floral organs.

Soluble proteins are important osmoprotectants and metabolic regulators. Their concentrations peaked in the I1 stage under all three treatments and declined thereafter, indicating intensive synthesis of enzymes and regulatory proteins during floral induction, followed by nitrogen remobilization to developing reproductive organs. Soluble protein contents declined with the rise in temperature before the I2 stage. This may reflect reduced bulk protein synthesis or altered protein turnover, which is consistent with the experimental results obtained in broccoli by Šola et al. [44]. From I3 to F2, however, T3 maintained relatively high soluble protein levels, together with enhanced expression of the *HSF*, *WRKY*, and *ERF* genes. These proteins may include heat-shock proteins, antioxidant enzymes, and other cytoprotective components.

Antioxidant responses also varied with the developmental stage. SOD activity increased from the vegetative stage to F1 and was the highest under T3, indicating that SOD participates in both rapid inflorescence differentiation and higher-temperature response in leaves. POD catalyzes phenol (PhOH) oxidation to yield phenoxy radicals (PhO·), while hydrogen peroxide is reduced to H_2_O after accepting electrons [45]. In this study, POD activity decreased in I1, increased from the I1 to I2 stages, and differed among treatments depending on the developmental stage. This variability implies that SOD-derived H_2_O_2_ was not removed exclusively by POD and may also have functioned as a developmental and temperature signal. After 10 d of treatment, peroxisome pathway genes including *PEX*, *MVP*, and *EH* were markedly upregulated in T3, which indicates that organelle remodeling and ROS homeostasis modulation occur prior to the late-stage peak of SOD activity.

The increased plant height under treatment T3 is largely consistent with the coordinated reprogramming of the auxin, gibberellin, and brassinosteroid signaling pathways. Aux/IAA, ARF, and SAUR proteins serve as central participants within the auxin signaling cascade for integrating temperature cues into plant development [46]. The *SAUR* genes are auxin-responsive targets that may be stimulated by warm conditions [13]. The *AUX1*, *AUX*/*IAA*, and *ARF* genes displayed upregulated transcription in T1, whereas two SAUR homologs were induced under T3, which indicates that warming redirected auxin output to elongation rather than uniformly activating the pathway in the early stage of temperature response.

Different type-B and type-A *ARR* homologs favored different temperature treatments, consistent with functional specialization within these gene families. Type-B *ARR*s drive the transcription of cytokinin downstream targets, while type-A *ARR*s form an inducible negative feedback loop to constrain signal transduction [47,48]. Treatment T2 yielded the maximum number of flowers per individual plant, which indicates that cytokinin-mediated meristematic activity, carbon resource supply, and floral primordia initiation were more favorably coordinated under this treatment in ‘Zijiao’. In contrast, T3 promoted stem elongation and stress acclimation without further improving floral yield. Because *ARR* homologs displayed contrasting expression patterns, the T2 phenotype is more likely to depend on specific family members and tissue-specific regulation than on the uniform activation of the cytokinin pathway in the early stage of temperature response.

The ethylene, JA, and MAPK signaling pathways are likely involved in regulating development and early temperature response under high-temperature conditions. CTR1 kinases directly phosphorylate EIN2 proteins to block ethylene signal transduction [18]. In ‘Zijiao’, T3 induced elevated *ETR* expression, suppressed the negative regulatory kinase *CTR1*, and upregulated most *ERF1*/*2* homologs. This set of expression patterns may be involved in early signal transmission via the EIN2-EIN3/ERF axis [18]. JAZ proteins repress the COI1-JA-Ile signaling module and also function as JA-inducible negative-feedback regulators [49]. The stable overaccumulation of *CmJAZ1*-*like* in chrysanthemum suppresses the transcription of florigen-associated genes and delays floral transition [19]. Thus, the upregulated expression of three *JAZ* genes may activate ethylene signaling in leaves under T3.

The expression profiles of four candidate temperature-responsive TFs across distinct developmental stages demonstrate that transcriptional divergence detected in leaves in early transcriptomes does not persist as a steady state throughout the entire flowering cycle. Instead, temperature modulates the timing of transcriptional peak accumulation. *CmHSFA4* peaked earlier in I2 under T3 but in I3 under T1 and T2. A second increase was observed during later inflorescence development under T3. Such dynamic variations demonstrate that T3 seedlings trigger protein homeostasis and sustain these responses throughout development.

The correlation analyses showed that T3 and T2 were significantly positively correlated with *CmWRKY40* and *CmMYB* expression, respectively. *CmWRKY40* showed several expression peaks in the F1 and F4 stages under T3 in this study. MPK3 and MPK6 directly phosphorylate WRKY33 to remodel the expression of downstream metabolic and defense-related genes [50]. In the early stage of temperature response, the synchronized transcriptional activation of *MEKK*, *MKK*, *MPK*, and *WRKY* may serve as the early molecular foundation for the subsequent responses of antioxidant enzymes and peroxisome remodeling.

In Arabidopsis, *MYB30* participates in oxidative and heat responses via *ANNEXIN*-mediated Ca^2+^ signaling and can accelerate *FT* expression and flowering specifically in phloem tissues [51,52]. *CmMYB* expression peaked in I1 and F3 under T2 in leaves, coinciding with rapid bud enlargement from F3 to F5. This correlation indicates that *CmMYB* may participate in coordinating sugar signaling and florigen pathways under T2 conditions, whereas its functions in early temperature responses in the ‘Zijiao’ cultivar remain to be further verified.

GNC/CGA1-type *GATA* transcription factors coordinate chlorophyll biosynthesis, starch accumulation, and nitrogen assimilation while directly repressing *SOC1* to delay flowering [53,54]. *CmGATA8* was expressed at relatively low levels during early development but showed a pronounced peak in F4 under T3 and a weaker increase under T2. The late *CmGATA8* peak under T3 may contribute to maintaining chlorophyll levels, soluble protein accumulation, and carbon–nitrogen balance while simultaneously restraining reproductive progression. Its correlation with the prolonged F4 phase indicates that *CmGATA8* may participate in delayed inflorescence development or play an important role in a compensatory response to prolonged thermal exposure. Although *Actin* was selected based on its previous use in chrysanthemum under temperature-related and developmental conditions [36,37], and satisfactory primer amplification efficiency and specificity were confirmed in the present study, its expression stability was not evaluated against additional candidate reference genes across the temperature treatments and developmental stages. Therefore, future studies should validate multiple reference genes under the same experimental conditions to further strengthen quantitative expression analyses.

## 5. Conclusions

Temperature strongly regulated floral transition, developmental progression, plant morphology, physiological metabolism, and gene expression in ‘Zijiao’. T1 slowed inflorescence development despite the early accumulation of soluble sugars and proteins. Among the three temperature regimes evaluated, T2 (25/16 °C) shortened the overall period of floral bud differentiation, particularly the floret differentiation phase, and was associated with the greatest floral-bud size and flower number in ‘Zijiao’. Under these experimental conditions, a comparatively favorable coordination among pigment content, floral developmental progression, and stage-specific transcriptional regulation was observed after treatment T2. T3 maintained vegetative growth, promoted stem elongation, induced the highest SOD activity and soluble protein content in the F1 stage, and showed a significant relationship with *CmWRKY40* expression. Additionally, the RNA-seq results obtained on the 10th treatment day merely mirror the preliminarily determined molecular events preceding phenotypic formation instead of reflecting steady transcriptional profiles across the entire flowering period. To verify these hypotheses in future studies, we will analyze the expression patterns of flowering regulators such as *FTL2*/*3*, *SOC1*, and floral meristem identity genes; investigate the change patterns of H_2_O_2_ and MDA; and integrate the verification of DELLA and HSF protein stability during floral bud differentiation mediated by temperature. Given the cultivar dependence of temperature responses in chrysanthemum, the relative advantage of 25/16 °C observed in this study should be validated in additional cultivars and production environments before broader application.

## Figures and Tables

**Figure 1:**
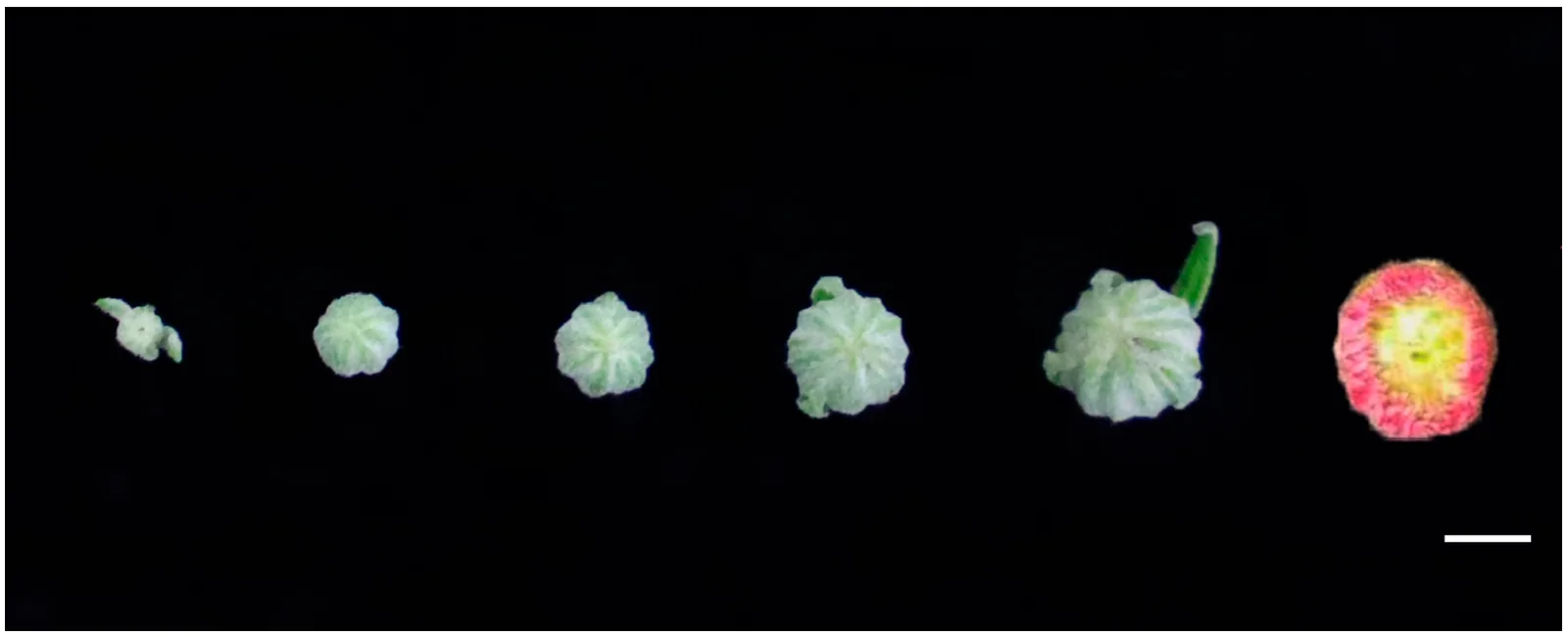
Developmental stages of chrysanthemum floral buds from bud emergence to visible flower coloration. Bar 1 cm.

**Figure 2:**
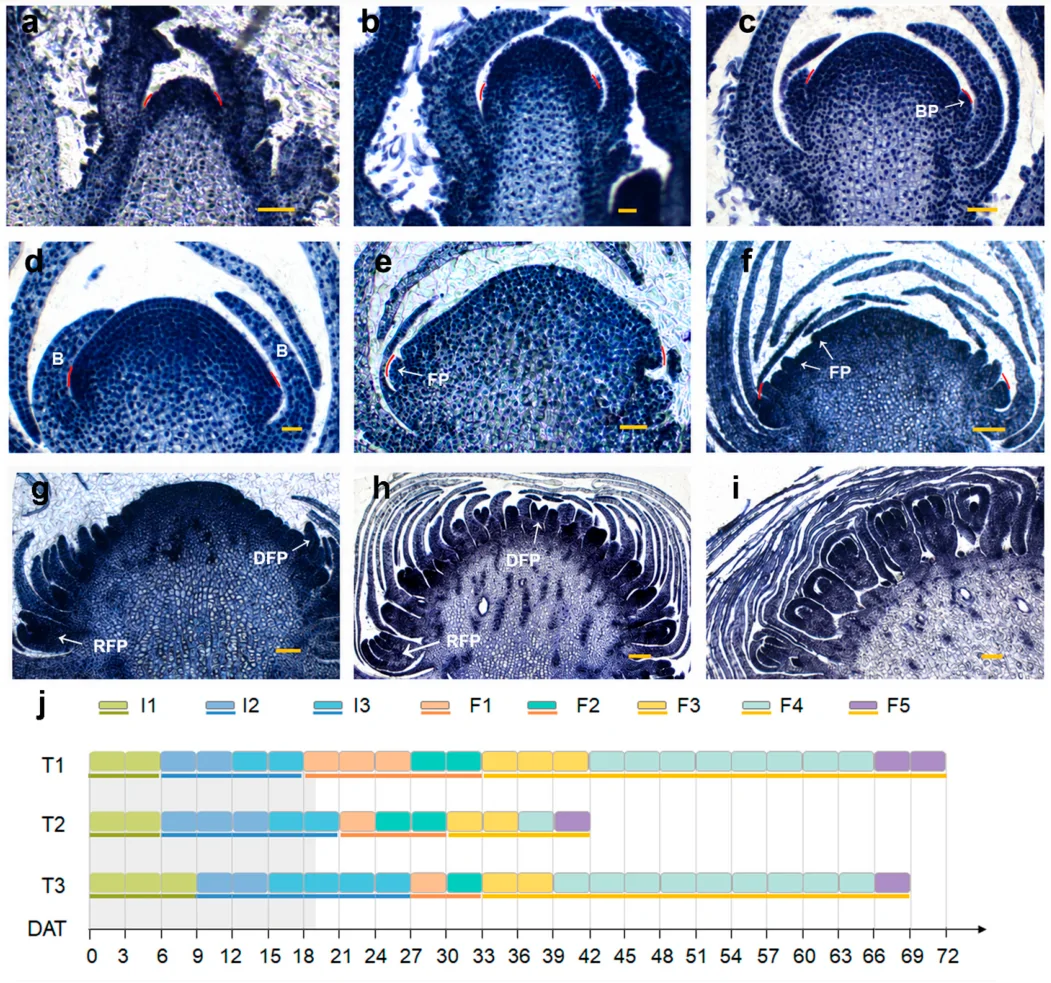
The anatomical structures of the SAM during floral bud differentiation (**a**–**i**) and the durations of these stages after different temperature treatments in chrysanthemum (**j**). (**a**) Vegetative growth (VJ) stage. (**b**) Inflorescence primordium differentiation (I1) stage. (**c**,**d**) Initial/final stages of involucre formation (I2/I3). (**e**,**f**) The initial/final floral primordium differentiation (F1/2) stages. (**g**–**i**) Initial/middle and final stages of corolla formation (F3/4/5). Short red arc lines mark the lateral boundaries of the meristem. BP, bract primordium; B, bract; FP, floret primordium; DFP, disk floret primordium; RFP, ray floret primordium. Bar 0.2 mm. (**j**) DAT, days after transplanting. T1, T2, and T3 represent temperature treatments of 20/13 °C, 25/16 °C, and 30/18 °C. The distinct colors of the blocks represent the eight different stages of floral bud differentiation. The horizontal lines below the blocks represent four periods, including the inflorescence primordium differentiation stage, the involucre formation stage, floral primordium differentiation, and the corolla formation stage. The shaded bars in chart represent the duration of the temperature treatments.

**Figure 3:**
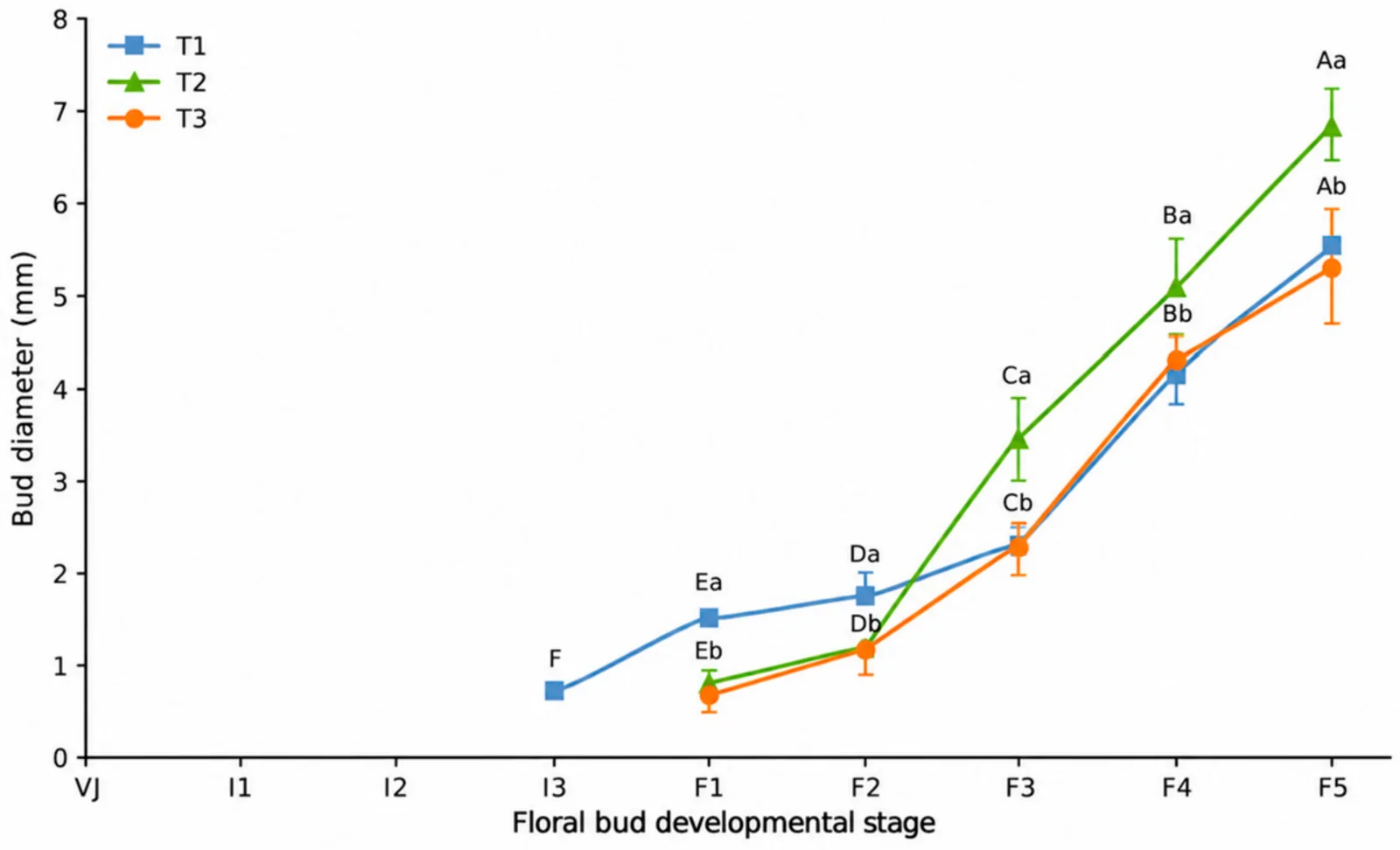
The average floral-bud diameter at different developmental stages during and after temperature treatments. Values represent means ± standard deviation (SD), with *n* = 10 biological replicates. Temperature, developmental stage, and their interaction were therefore evaluated over F1–F5 using repeated-measures ANOVA. The I3 value under T1 was not included in the between-temperature factorial analysis. Uppercase letters indicate differences among developmental stages within the same temperature treatment, whereas lowercase letters indicate differences among temperature treatments within the same developmental stage (*p* < 0.05). The I3 value under T1 was not included in the between-temperature factorial analysis.

**Figure 4:**
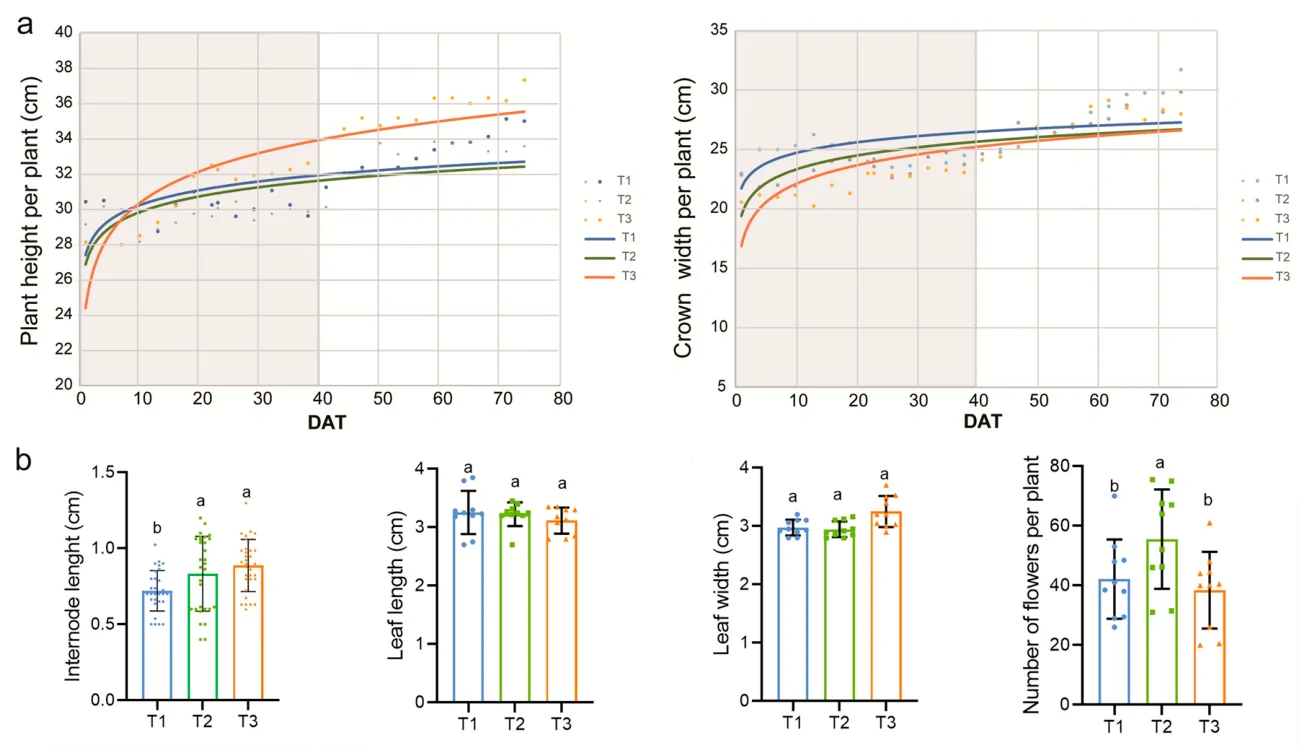
Phenotypic characteristics of chrysanthemum plants during and after different temperature treatments. (**a**) Fitted growth curves for plant height and crown width during flowering. DAT, days after transplanting. The shaded bars in chart represent the duration of the temperature treatments. (**b**) Internode length, leaf length, and leaf width measured at full bloom in plants returned to the field after different temperature treatments. Data in panel (**b**) are presented as the means ± SD of 10 biological replicates. One-way ANOVA was used to analyze significant differences among different temperature treatments at full bloom. Statistical significance was determined with a *p* < 0.05. Lowercase letters indicate between-group differences.

**Figure 5:**
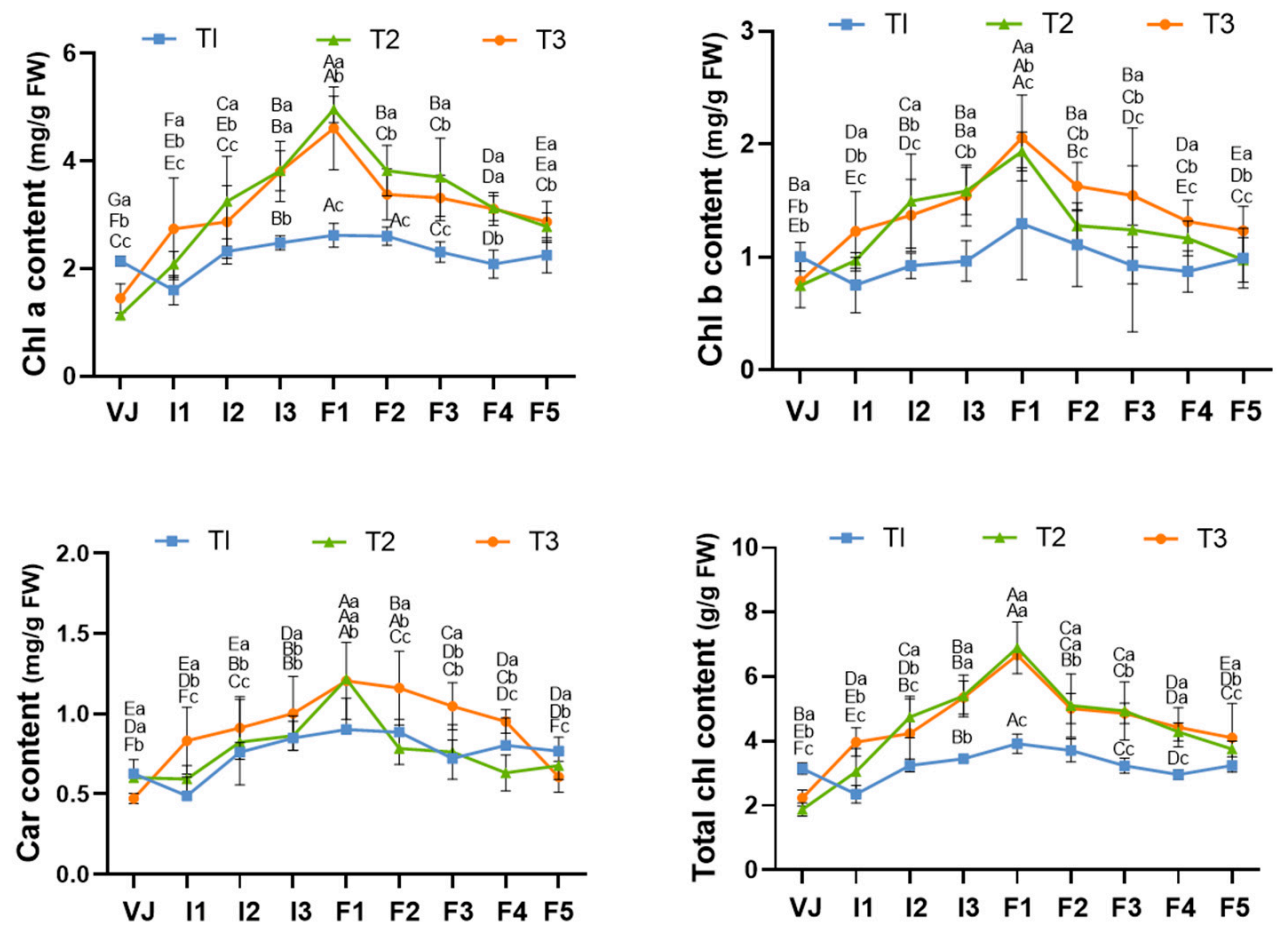
Changes in photosynthetic pigment contents during floral bud differentiation in ‘Zijiao’ during and after different temperature treatments. Data are presented as means ± SD. The effects of temperature treatment, developmental stage, and their interaction were evaluated by two-way ANOVA. When a significant interaction was detected, simple-effect comparisons were performed among temperature treatments within each developmental stage and among developmental stages within each temperature treatment. Lowercase letters indicate differences among temperature treatments within the same developmental stage, whereas uppercase letters indicate differences among developmental stages within the same temperature treatment (*p* < 0.05).

**Figure 6:**
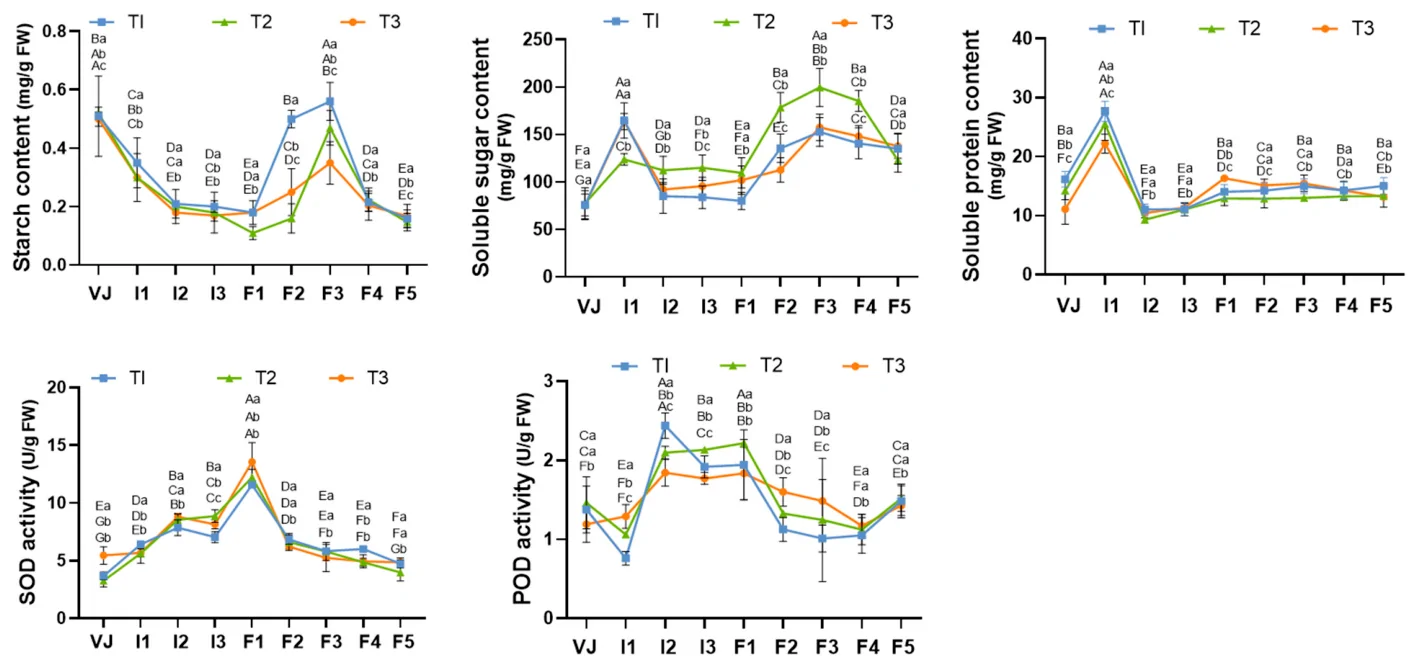
Changes in carbohydrate and soluble protein contents and antioxidant enzyme activity during floral bud differentiation in ‘Zijiao’ under different temperature treatments. Data are means ± SD (*n* = 3). The effects of temperature treatment, developmental stage, and their interaction were evaluated by two-way ANOVA. Where appropriate, simple-effect comparisons were conducted following the factorial analysis. SOD and POD represent superoxide dismutase and peroxidase, respectively. Lowercase letters indicate differences among temperature treatments within the same developmental stage, whereas uppercase letters indicate differences among developmental stages within the same temperature treatment (*p* < 0.05).

**Figure 7:**
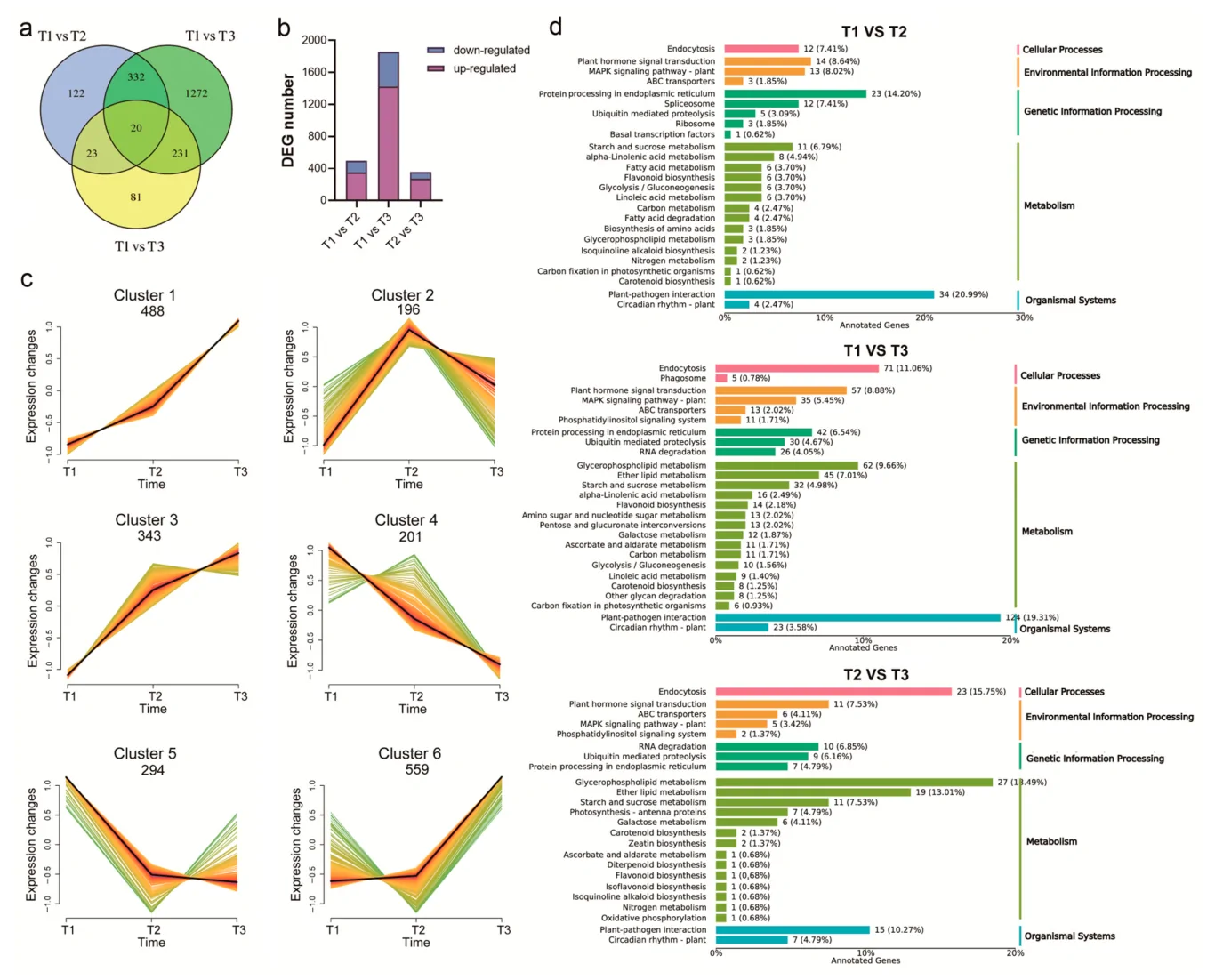
DEG expression patterns and KEGG pathway classification in chrysanthemum under different temperature treatments. (**a**) Venn diagram of the significantly DEGs in different com-treatment pairs. (**b**) The number of significantly DEGs. (**c**) The proximity of DEGs to the cluster center, where black indicates the closest proximity, yellow represents intermediate proximity, and green shows the farthest proximity. (**d**) KEGG pathway classification of the DEGs identified in each pairwise comparison.

**Figure 8:**
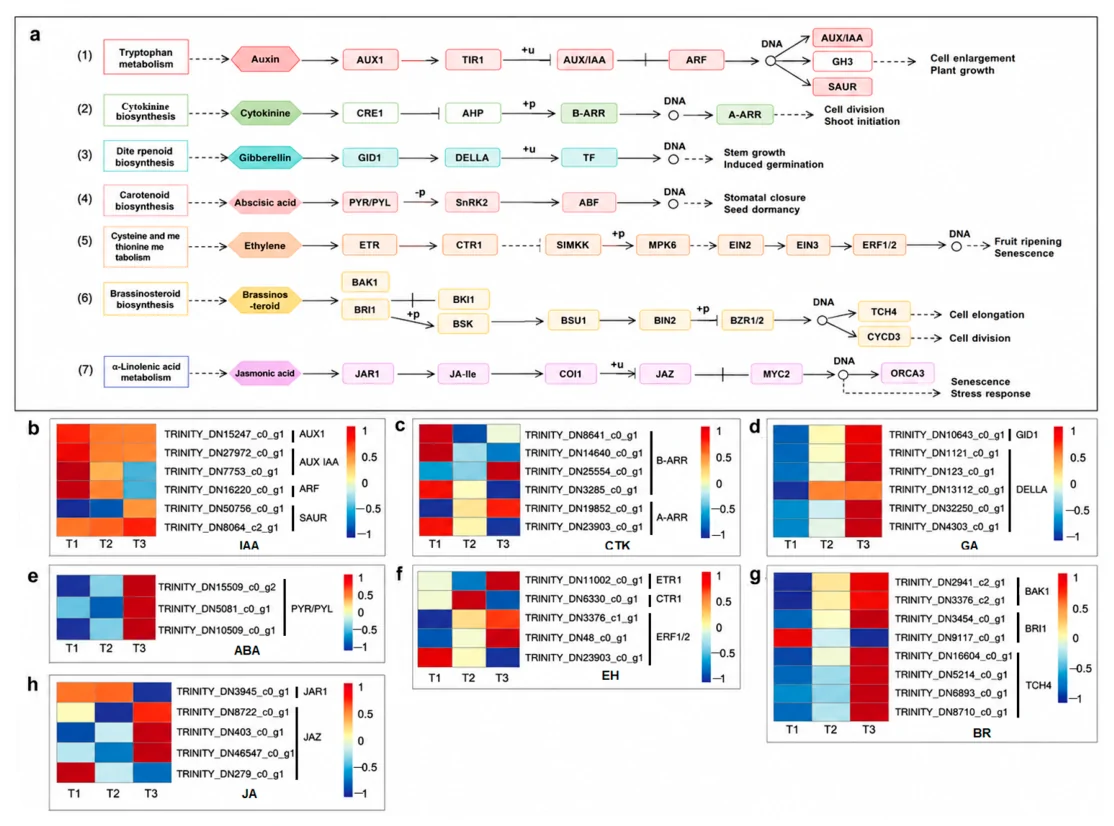
Temperature-responsive expression patterns of genes related to plant hormone signal pathways in chrysanthemum. (**a**) Schematic diagram of the plant hormone signaling pathway, with the key coding genes involved shaded. Solid arrows indicate direct or defined regulatory relationships, whereas dashed arrows indicate indirect relationships or omitted intermediate steps. (**b**–**h**) Heatmap of DEGs in different gene modules.

**Figure 9:**
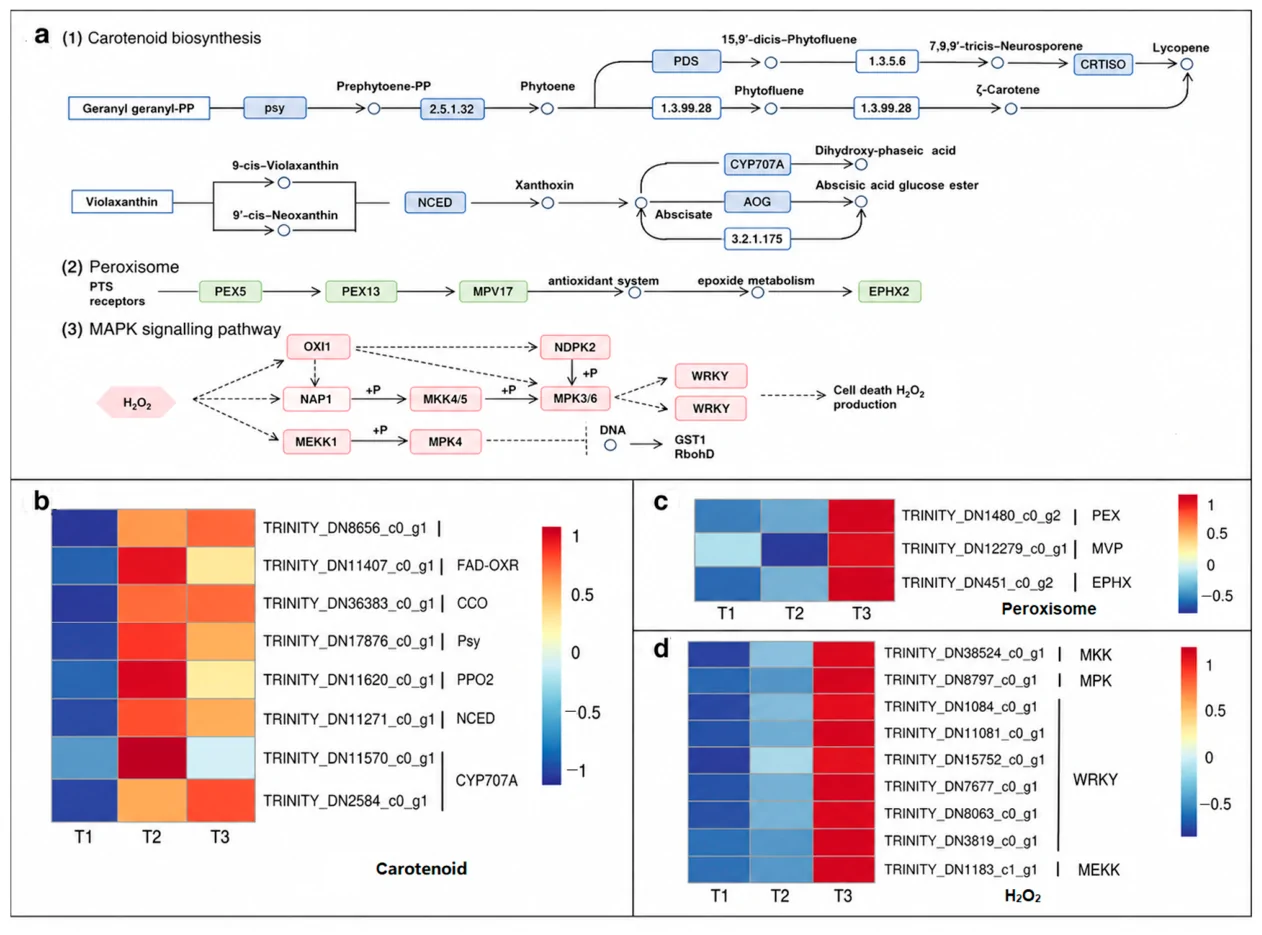
Identification and expression profiles of temperature-responsive gene modules associated with physiological traits in chrysanthemum. (**a**) Schematic representation of regulatory cascade linking temperature-responsive DEGs to changes in physiological traits under different temperature treatments. Solid arrows indicate direct or defined regulatory relationships, whereas dashed arrows indicate indirect relationships or omitted intermediate steps. (**b**–**d**) Heatmaps of DEGs in different gene modules.

**Figure 10:**
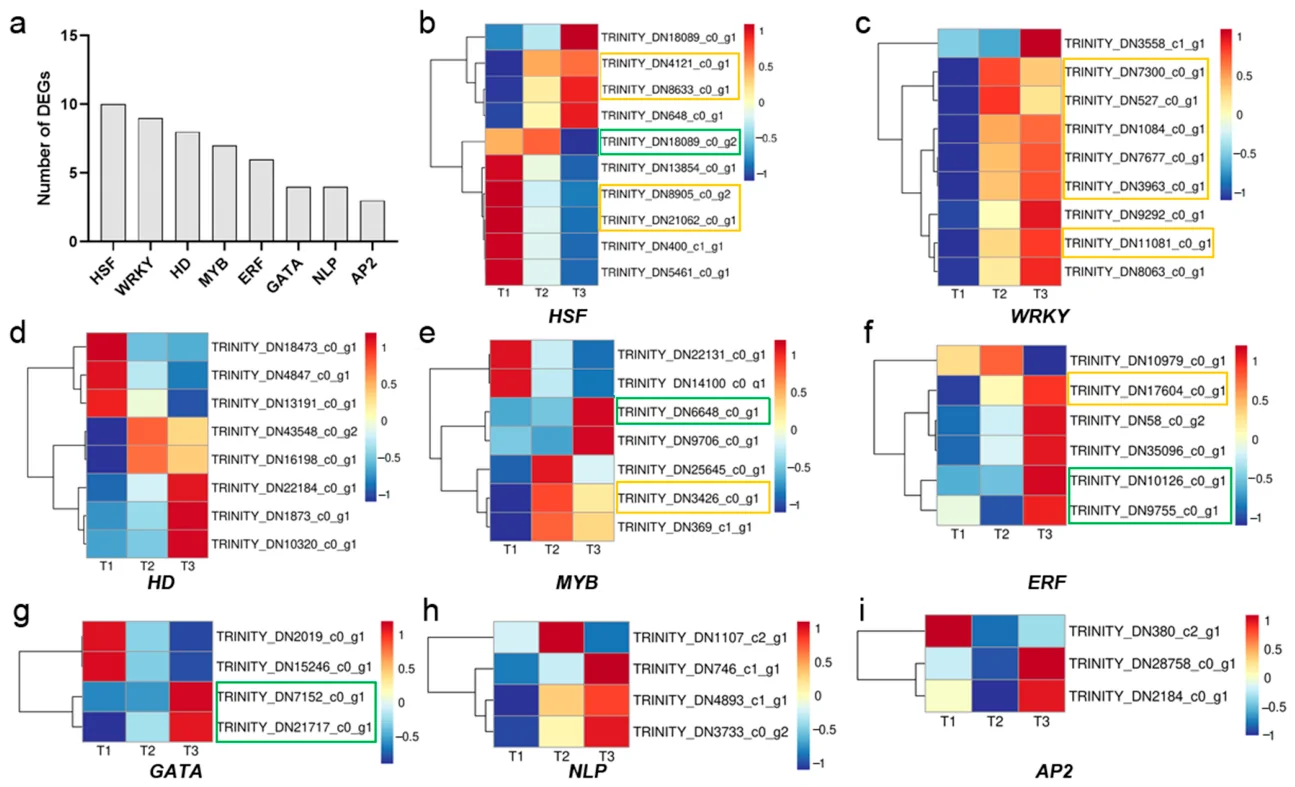
Identification and expression profiles of temperature-responsive transcription factors during ‘Zijiao’ floral bud differentiation. (**a**) Categorization of transcription factor families in response to temperature. (**b**–**i**) Heatmaps illustrating the DEGs encoding *HSF* (**b**) and *WRKY* (**c**) transcription factors. The yellow boxes indicate transcription factors shared by the T1 vs. T2 and T1 vs. T3 pairs, whereas the green boxes indicate transcription factors shared by the T2 vs. T3 and T1 vs. T3 pairs.

**Figure 11:**
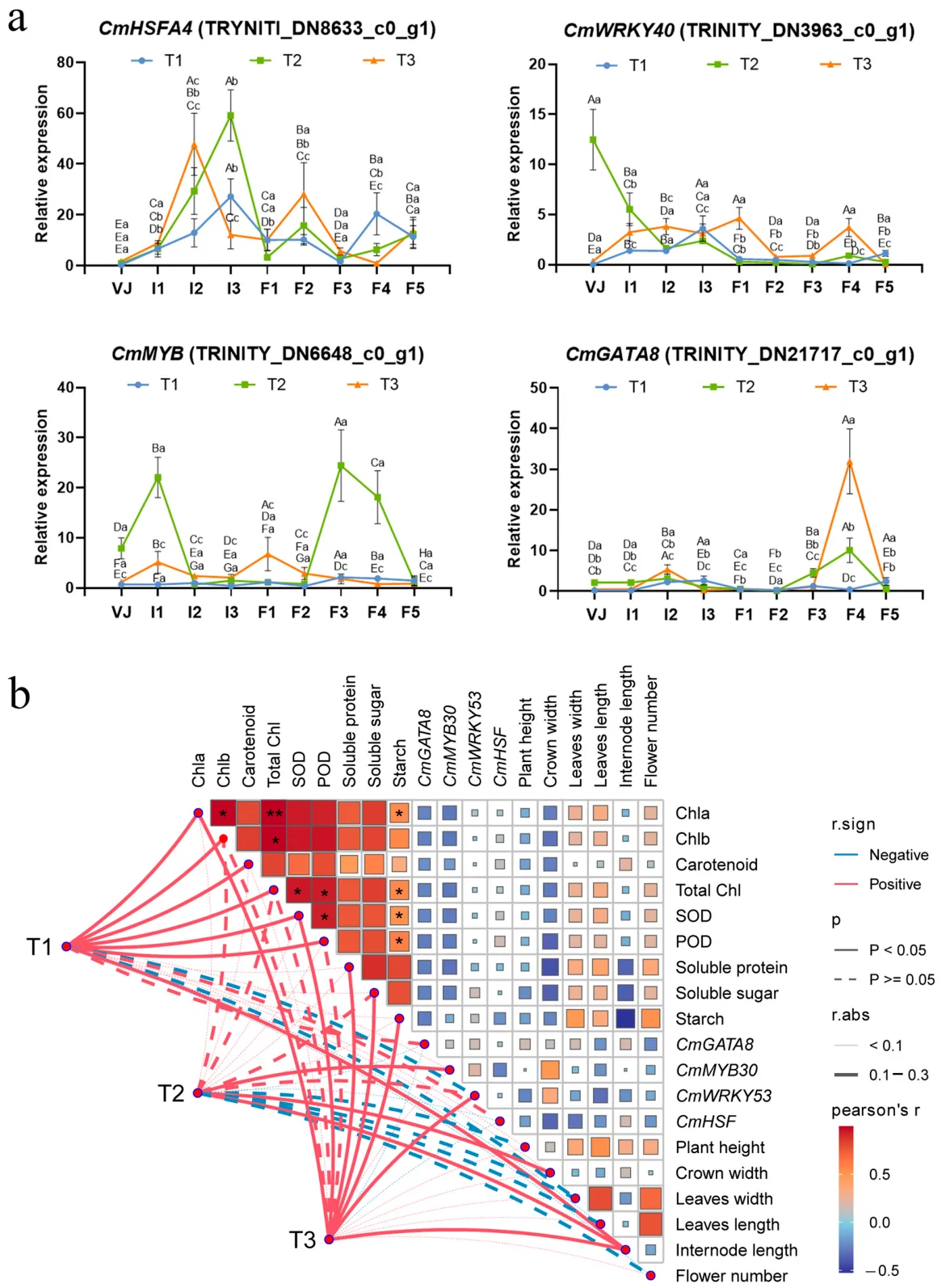
Expression profiles of selected temperature-responsive TFs during floral bud differentiation in ‘Zijiao’ and their correlations with other parameters. (**a**) Developmental expression patterns of candidate TFs. Data are presented as means ± SD. The effects of temperature treatment, developmental stage, and their interaction were evaluated using two-way ANOVA. (**b**) The Advanced Cor link analysis based on all indices. Pairwise comparisons of morphological, physiological, and biochemical indicators and the expression patterns of candidate temperature-responsive TFs, with the color gradient denoting Pearson correlation coefficients. Mantel tests were used to assess the correlations between the three temperature regimes and the indices. Here, *r* denotes Pearson’s correlation coefficient; r.sign and r.abs indicate the sign and absolute value of *r*, respectively, and P denotes the *p* value. Pearson correlation values that are significantly different at *p* < 0.05 (*) or *p* < 0.01 (**) are indicated. Edge width corresponds to the Mantel r statistic for the corresponding distance correlations. The different size boxes reflect the absolute value of *r*.

## Data Availability

The datasets generated during the current study are available in the NGDC GSA (Genome Sequence Archive, https://ngdc.cncb.ac.cn/gsub/), under the accession number CRA032422.

## Supplementary Materials

The following supporting information can be downloaded at: https://www.mdpi.com/article/10.3390/life16101600/s1, Figure S1: Field air temperature monitoring after transplanting ‘Zijiao’ to the field; Figure S2: Specificity of PCR of each quantitative primer pair; Figure S3: Sample reproducibility and correlation analysis principal component analysis (PCA) plot; Figure S4: RT-qPCR validation of candidate temperature-responsive TFs at day 10; Table S1: The reaction system of specificity of RT-qPCR; Table S2: The reaction condition of specificity for RT-qPCR analysis; Table S3: Specific RT-qPCR primers of ‘Zijiao’ and their amplification characteristics; Table S4: The duration of floral bud differentiation in ‘Zijiao’ during and after different temperature treatments; Table S5: Effects of temperature treatment, developmental stage, and their interaction on morphological and physiological traits during floral bud differentiation; Table S6: Summary statistics of sequencing data for samples; Table S7: Summary statistics of the assembly results; Table S8: Effects of temperature treatment, developmental stage, and their interaction on candidate transcription-factor expression during floral bud differentiation.
