# Supplementary material for: Physiological and Transcriptomic Analyses Reveal Divergent Responses of Vegetative Growth and Inflorescence Development to Temperature in Chrysanthemum × morifolium
Source: Life (Basel). 2026 Sep 24;16(10):1600. doi: 10.3390/life16101600 (PMC13706349; doi:10.3390/life16101600)
Supplement: Supplementary file 1 [file life-16-01600-s001.zip › life-4506880-supplementary.pdf]

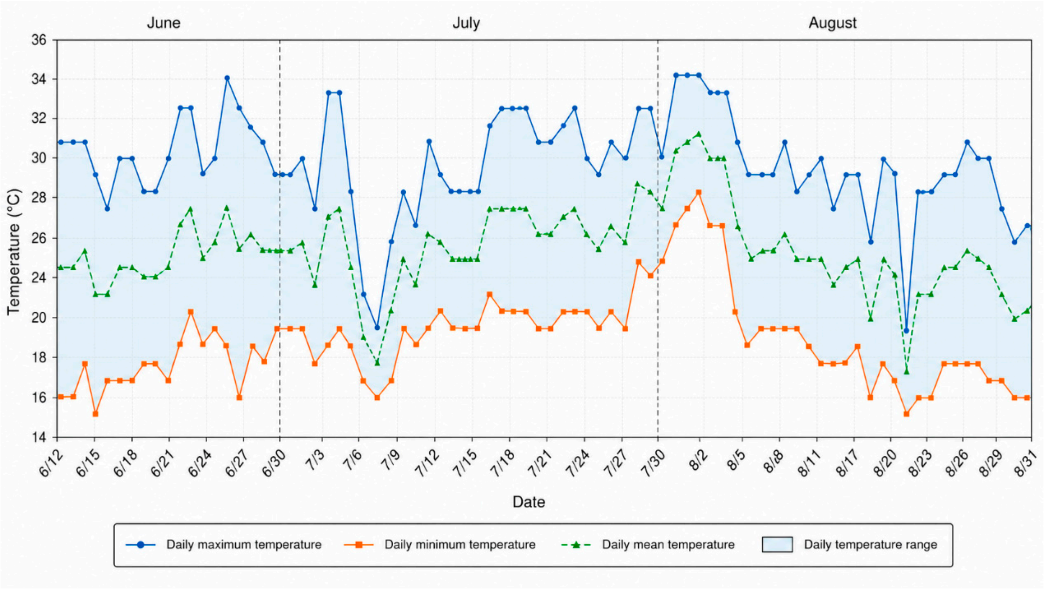

Figure S1. Field air temperature monitoring after transplanting 'Zijiao' to the field.

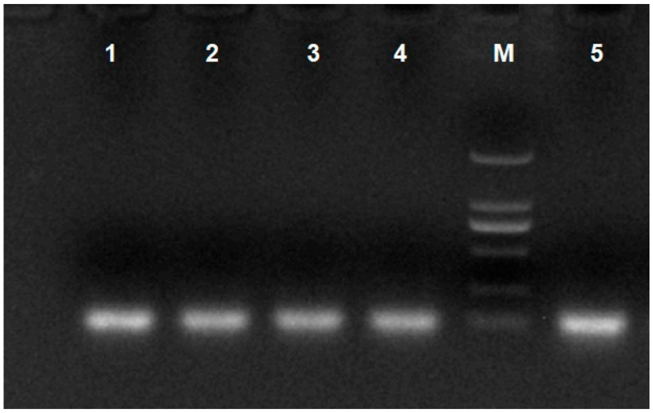

Figure S2. Specificity of PCR of each quantitative primer pair. M represents the DL2000 DNA marker. 1-5 represent the quantitative primer pair of *CmHSFA4*, *CmGATA8*, *CmMYB*, *CmWRKY40* and *Actin*, respectively.

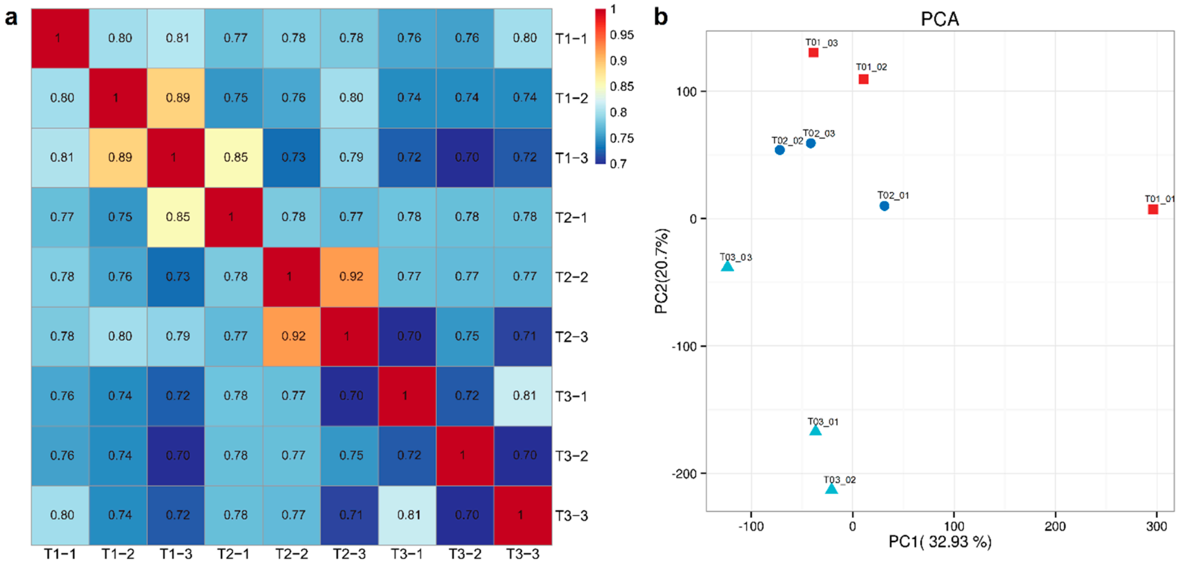

Figure S3. Sample reproducibility and correlation analysis principal component analysis (PCA) plot. (a) Inter-sample correlation heatmap. (b) PCA analysis.

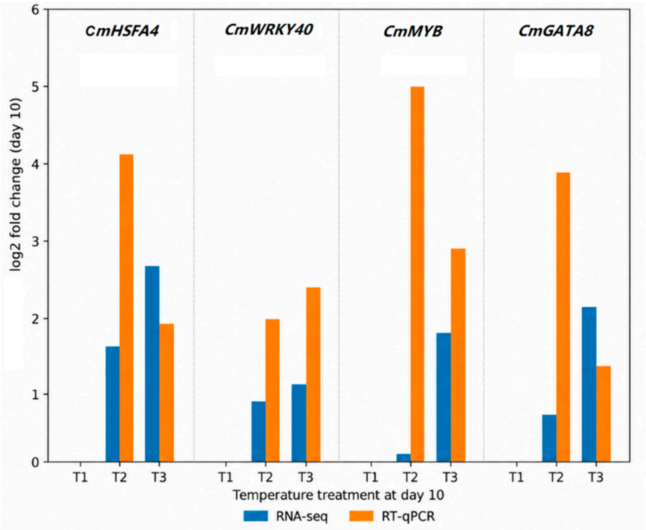

Figure S4. RT-qPCR validation of candidate temperature-responsive TFs at day 10.

Table S1. The reaction system of specificity of RT-qPCR.

Component	Volume（μL）
SYBR qPCR Mix Without Rox	10.0
F/R Primer (10μM)	0.4
Template	0.2
ddH2O	9.0

Table S2. The reaction condition of specificity for RT-qPCR analysis.

95℃	2min	
95℃	10s	40 cycles
Tm	15s	
72℃	30s	
72℃	2min	

Table S3. Specific RT-qPCR primers of 'Zijiao' and their amplification characteristics.

name	Primer Sequence (5'-3')	Primer efficiency	R^2
Actin F	CCAAAAGCCAATCGTGAGAAG	107.3%	0.997
Actin R	CACCATCACCAGAATCCAACA		
CmHSFA4 F	AATGAGGATTTTATAAGAGG	92.1%	0.996
CmHSFA4 R	AGTACTAATCAAGTTTGCCC		
CmGATA8 F	TTTCCCCTTCAACCCTCGTC	115.9%	0.996
CmGATA8 R	TTGAATGGAGCGTCGGGTTT		
CmMYB F	AACCCTCCACACAAACACCT	100.4%	0.998
CmMYB R	CTTCACCACTCGACTCACGA		
CmWRKY40 F	ACCCCGTGTCAGTCGAGATT	94.2%	0.992
CmWRKY40 R	TTAGCCAGCCTGAGTCATCG		

Table S4. The duration of floral bud differentiation in 'Zijiao' during and after different temperature treatments.

Treatments	Days before each bud differentiation period during and after temperatures (d)									
	VJ	I1	I2	I3	F1	F2	F3	F4	F5	The total days of floral bud differentiation duration (d)
T1	0	6±1	12±1	18±1	27±1	33±1	42±1	66±2	72±3	72±3
T2	0	6±1	15±1	21±1	24±1	27±1	33±1	36±1	42±1	42±1
T3	0	9±1	15±1	27±1	30±2	33±1	39±1	66±1	69±2	69±2

Table S5. Effects of temperature treatment, developmental stage, and their interaction on morphological and physiological traits during floral bud differentiation.

Trait	Stages included	Temperature	Developmental stage	Temperature× Stage
Floral-bud diameter	F1–F5	F = 17.31, p <0.001	F = 21.70, p < 0.001	F = 3.41, p < 0.001
Chl a	VJ–F5	F = 18.71, p <0.001	F = 31.40, p <0.001	F = 4.12, p <0.001
Chl b	VJ–F5	F = 15.20, p <0.001	F = 18.81, p <0.001	F = 3.36, p <0.001
Carotenoids	VJ–F5	F = 22.60, p <0.001	F = 16.90, p <0.001	F = 4.85, p <0.001
Total chlorophyll	VJ–F5	F = 20.90, p <0.001	F = 29.77, p <0.001	F = 4.37, p <0.001
Starch	VJ–F5	F = 19.42, p <0.001	F = 37.60, p <0.001	F = 1.54, p=0.120
Soluble sugar	VJ–F5	F = 11.70, p <0.001	F = 24.30, p <0.001	F = 3.21, p <0.001
Soluble protein	VJ–F5	F = 8.97, p <0.001	F = 17.50, p <0.001	F = 4.04, p <0.001
SOD activity	VJ–F5	F = 31.52, p <0.001	F = 26.20, p <0.001	F = 1.43, p=0.172
POD activity	VJ–F5	F = 7.82, p =0.001	F = 13.60, p <0.001	F = 2.83, p=0.172

Note: Temperature and developmental stage were treated as fixed factors. For independently sampled biological replicates, two-way factorial ANOVA was used. Significant temperature × developmental-stage interactions indicate that the magnitude or direction of the temperature response differed among developmental stages. For floral-bud diameter, the factorial analysis was restricted to F1 – F5 because these were the developmental stages at which measurable visible buds were present under all three temperature treatments.

Table S6. Summary statistics of sequencing data for samples.

ID	Read Number	Base Number	GC Content	%≥Q30
T1-1	26,521,992	7,934,947,156	44.57%	94.09%
T1-2	25,158,226	7,527,123,658	43.98%	93.98%
T1-3	25,153, 712	7,525,144,546	44.21%	93.89%
T2-1	27,318,844	8,182,319,042	44.55%	93.67%
T2-2	29,118,544	8,728,351,246	44.38%	93.66%
T2-3	28,383,146	8,449,783,612	44.45%	93.66%
T3-1	26,741,470	7,985,353,102	44.31%	93.53%
T3-2	26,643,223	7,970,936,186	43.77%	93.68%
T3-3	26,371,215	7,968,436,207	43.23%	93.56%
Total	216,256,660	72,272,394,755	-	-
Mean value	27,032,083	8,030,266,08	44.16%	-

Note: Read Number: Total number of pair-end reads in Clean Data; Base Number: Total number of bases in Clean Data; GC Content: GC content of Clean Data, i.e., the percentage of G and C bases in the total bases of Clean Data; %≥Q30: Percentage of bases with quality value greater than or equal to 30 in Clean Data.

Table S7. Summary statistics of the assembly results.

Length Range	Transcript	Unigene
200-300	35,376(17.76%)	21,090(27.82%)
300-500	47,207(23.70%)	19,371(25.55%)
500-1000	55,599(27.91%)	17,003(22.43%)
1000-2000	44,059(22.12%)	12,556(16.56%)
2000+	16,943(8.51%)	5,788(7.64%)
Total Number	199,184	75,808
Total Length	176,543,424	58,900,942
N50 Length	1,302	1,255
Mean Length	886.33	776.98

Note: Length Range, length intervals of Unigenes with numbers indicating counts and percentages in parentheses indicating proportions; Total Number, total number of assembled Unigenes; Total Length, total length of assembled Unigenes; N50 Length, N50 length of Unigenes; Mean Length, average length of Unigenes.

Table S8. Effects of temperature treatment, developmental stage, and their interaction on candidate transcription-factor expression during floral bud differentiation.

Variable	Temperature, F(2,54)	P	Stage, F(8,54)	P	Temperature × Stage, F(16,54)	*p*
CmHSFA4	16.40	<0.001	21.70	<0.001	4.66	<0.001
CmWRKY40	25.12	<0.001	28.90	<0.001	6.12	<0.001
CmMYB	18.90	<0.001	24.50	<0.001	5.31	<0.001
CmGATA8	29.80	<0.001	19.73	<0.001	4.27	<0.001

Note: Temperature and developmental stage were treated as fixed factors. For independently sampled biological replicates, two-way factorial ANOVA was used. Significant temperature × developmental-stage interactions indicate that the magnitude or direction of the temperature response differed among developmental stages.
